# Hydrochemical evolution and formation mechanism of groundwater affected by human activities in Zhangxuan basin, northwest of Yanshan mountains, China

**DOI:** 10.1371/journal.pone.0318995

**Published:** 2025-02-13

**Authors:** Miaomiao Ma, Yanbo Yu, Baizhong Yan, Yapeng Tuo, Junbai Gai

**Affiliations:** 1 Hebei Province Collaborative Innovation Center for Sustainable Utilization of Water Resources and Optimization of Industrial Structure, Hebei GEO University, Shijiazhuang, China; 2 Hebei Province Key Laboratory of Sustained Utilization & Development of Water Resources, Shijiazhuang, China; Yogi Vemana University, INDIA

## Abstract

The Zhangxuan Basin serves as an ecological barrier and water conservation area for Beijing, the capital of China. Clarifying the hydrochemical evolution of groundwater in this region is essential for the effective management of groundwater resources and the protection of ecological security. In this study, based on data regarding chemistry and level of shallow groundwater from 2016 to 2022, hydrogeochemical analysis methods and geostatistical techniques were applied to investigate the hydrochemical evolution and genesis mechanisms of groundwater under the influence of human activities. The results showed that: (1) From 2016 to 2022, the groundwater remained predominantly characterized by Ca^2+^ and HCO_3_^-^, with the primary hydrochemical type unchanged as HCO_3_-Ca·Mg. (2) From 2016 to 2022, an overall decreasing trend in hydrochemical components was observed, alongside an increase in HCO_3_-Na type groundwater. Spatially, along the direction of groundwater flow, a general trend of increasing hydrochemical components was noted, with a significant rise in HCO_3_-Na type groundwater. (3) The spatiotemporal distribution and evolution of hydrochemistry were influenced by water-rock interactions, lithological characteristics, groundwater flow patterns, and human activities. Along the groundwater flow direction, lithological particles became finer, enhancing forward cation exchange and leaching, with the dissolution of silicate and carbonate minerals intensifying. In localized areas, the hydrochemical components were influenced by the extraction of groundwater source areas and the discharge of industrial waste.

## 1 Introduction

Groundwater, a fundamental water source essential for human sustenance and existence, occupies a pivotal position within the global hydrological cycle [1,2]. It is regarded as a critical asset for societal and economic progress, while also contributing to ecological equilibrium. In recent years, economic development has induced changes in the chemical composition of groundwater due to industrial production and agricultural irrigation, leading to risks to human health [3–6]. Additionally, excessive exploitation and imprudent utilization of groundwater resources have resulted in a gradual depletion of groundwater level [7,8], which indirectly impacts the hydrochemical characteristics of groundwater. Zhangjiakou, located in the northwest region of Beijing, China, is situated within the upper reaches of the Yongding River basin and shares ecological interconnections with downstream Beijing. Its strategic geographical position and abundant natural resources render Zhangjiakou a significant ecological buffer and water conservation area for Beijing, playing a crucial role in safeguarding the capital’s water resources and ecological integrity [9]. Groundwater is the primary water supply for Zhangjiakou [10]. The hydrodynamic characteristics of groundwater have been altered by human activities, including industrial and agricultural practices, as well as groundwater extraction [11]. Consequently, the quality of groundwater has been compromised, posing risks to the safety of the water environment. Therefore, research on the hydrochemical evolution and genesis mechanisms of groundwater is essential for protecting the local ecological environment and establishing a robust ecological shield for the capital city.

Numerous studies have extensively investigated the evolution and genesis mechanisms of hydrochemistry. Two commonly utilized methods for examining groundwater chemistry evolution are multivariate statistical analysis and hydrogeochemical analysis. These methods encompass various techniques, including the Piper diagram, Gibbs diagram, rock weathering end-member diagram, chlor-alkali index diagram, geostatistical analysis, and ion ratio relationship analysis [12,13]. The Piper diagram facilitates visual observation of hydrochemical types, aiding in the study of groundwater chemical evolution characteristics and the origins of soluble chemical components [14]. The Gibbs diagram is employed to analyze the natural evolution mechanism of groundwater chemistry and to identify the controlling factors influencing chemical components [15–17]. Pearson correlation analysis is utilized to demonstrate relationships between different variables. For example, Mohamed et al. [18] evaluated water-rock interactions in southern Egypt using methods such as the chlor-alkali index, correlation analysis, and the Gibbs diagram. Extensive investigations into the spatial distribution characteristics of hydrochemistry and the origins of ions can be conducted using geostatistical techniques and ion ratio correlations [19–21]. For instance, Boudibi et al. [22] assessed and projected groundwater chemical parameter concentrations in southeastern Algeria by employing ordinary kriging and other methods. Similarly, Zhan et al. [23] investigated the evolutionary mechanism of groundwater chemistry in rural northern China in the context of chemical plant wastewater discharge, utilizing techniques such as multivariate statistics and hydrogeochemistry. Ha et al. [24] identified significant impacts of human activities on groundwater in southern Vietnam by employing the WQI and multivariate statistical analysis. Furthermore, Barmakova et al. [25] investigated the influence of agricultural irrigation on groundwater chemical characteristics in southeastern Kazakhstan using the sodium adsorption ratio method, geostatistical analysis, and spatial interpolation techniques. Thus, globally, research on factors influencing the chemical evolution of groundwater often focuses on natural geological settings and human activities. In recent years, attention in China has shifted towards understanding the relationship between groundwater level and hydrochemistry, offering new insights into the global study of groundwater chemical evolution and its causes. For example, Chen et al.[26] analyzed the hydrochemical evolution characteristics and mechanisms of groundwater in the Hengshui funnel area of China, both before and after comprehensive management, using groundwater level and hydrochemical data. Additionally, Li et al. [27] examined the hydrodynamic and isotopic characteristics and spatial distribution of Chagan Lake and groundwater by utilizing groundwater level, hydrochemistry, and stable hydrogen-oxygen isotope data.

The hydrochemical evolution and formation of groundwater are influenced by various factors. These factors are affected not only by the original geological environment of the groundwater but also by elements such as groundwater flow and human interventions [28]. Furthermore, these influencing factors are interconnected and collectively determine the spatial and temporal distribution and development of groundwater chemistry. In this study, based on the data of groundwater chemistry and levels of shallow groundwater from 2016 to 2022, methods such as Piper diagram, Gibbs diagram, chlor-alkali index diagram, ion ratio relationship analysis, correlation analysis, and geostatistical analysis were employed to investigate the hydrochemical evolution and genesis mechanisms of groundwater influenced by both natural factors and human activities. The results provide valuable theoretical support for groundwater pollution prevention and environmental protection in Zhangjiakou.

## 2 Materials and methods

### 2.1 Study area

**Fig 1:**
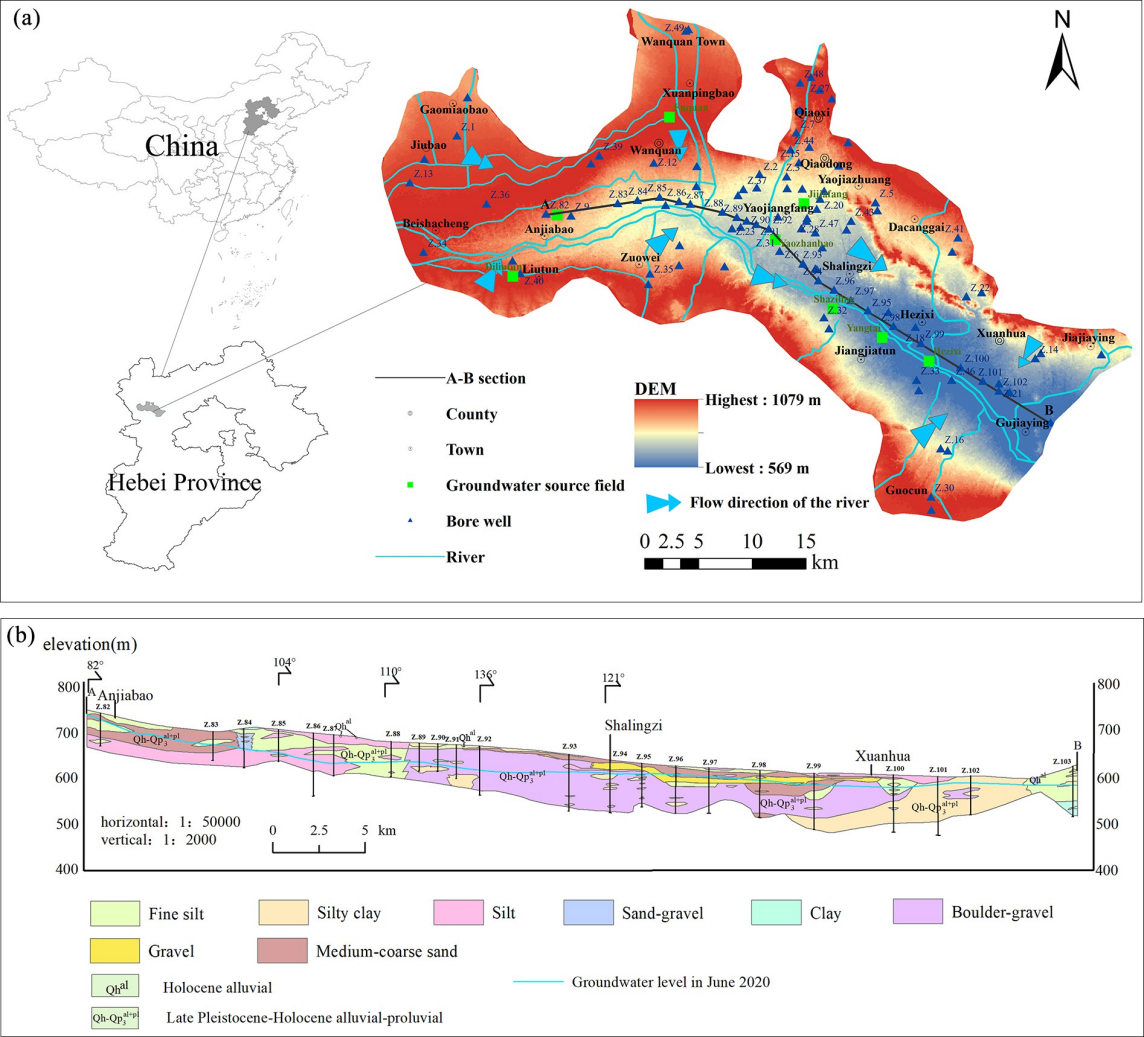
Sampling point distribution(a) and stratigraphic section(b) in the study area.

The study area is located in the central region of Zhangjiakou City, northwest Hebei Province (Fig 1A). It encompasses the central urban districts (Qiaodong District, Qiaoxi District), as well as Xuanhua District and Wanquan District. This region serves as the central hub for social and economic activities in Zhangjiakou, despite its fragile ecological environment. It is bordered by surrounding counties, including Zhangbei, Chongli, Chicheng, Huailai, and Zhuolu. The area spans geographical coordinates from approximately 114°09’42.76" to 115°19’53" east longitude and from 40°26’58" to 40°57’38" north latitude, covering approximately 1942.855 km^2^. The study area is situated in the northern cold temperate continental monsoon climate zone. It has an average annual temperature of 5.7°C and a multi-year average precipitation of 370.73 mm. Precipitation mainly occurs from June to September, while the average annual water surface evaporation is 1768.65 mm. This area is part of the Yongding River system within the Hai River basin, with the Yang River and its tributaries as the main waterways. The Yang River flows 106 km from west to east, converging with the Sanggan River at Jiahe Village in Huailai County. It then continues eastward for 10.5 km, entering the Guanting Reservoir before merging with the Yongding River. The study area is dominated by agricultural industries such as animal husbandry, vegetables, potatoes, and maize. Industrial activities mainly involve energy, mining, metallurgy, machinery, and food processing.

**Fig 2:**
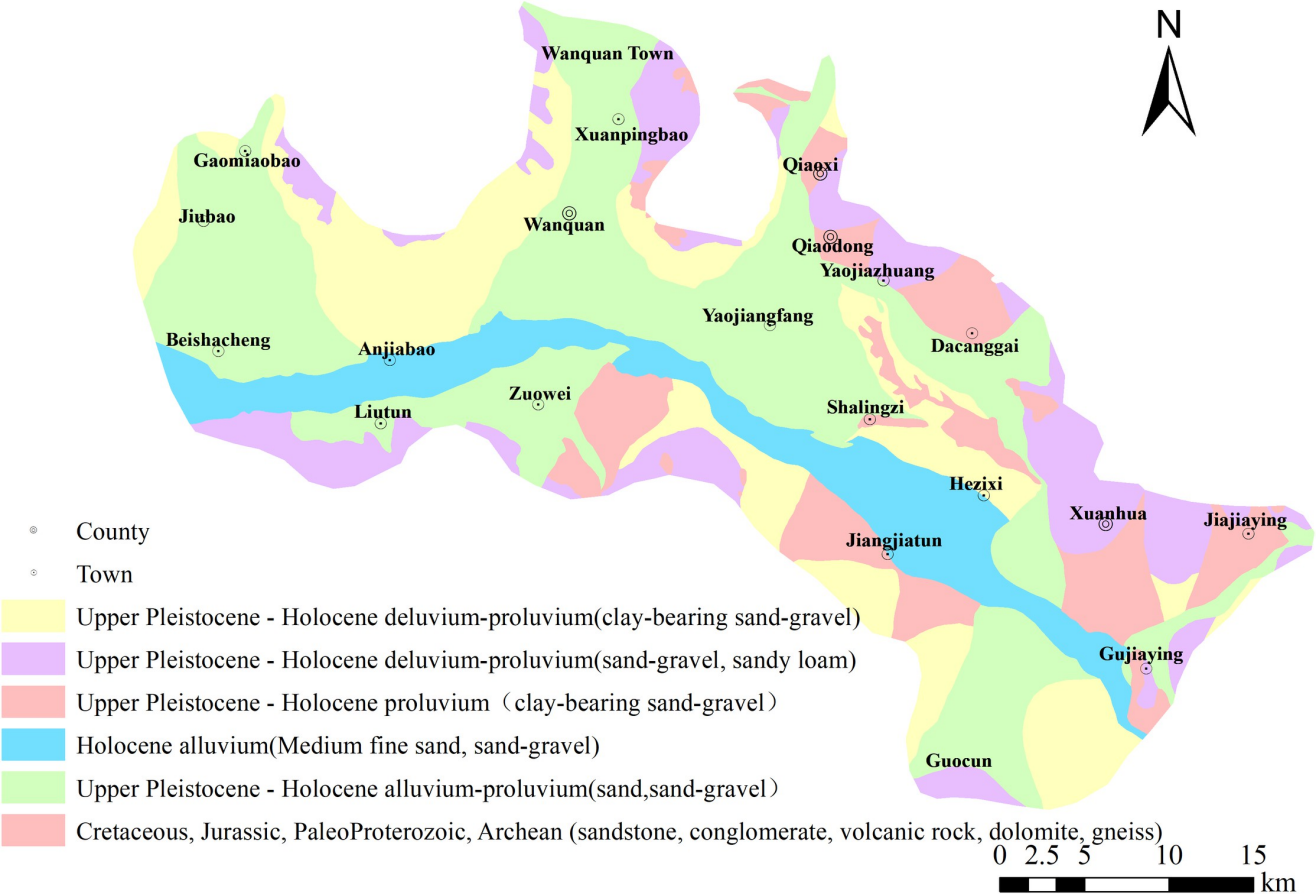
The geological formation of the study area.

The research area is an intermountain basin located in the western section of the Yanshan Mountains in northwest Hebei. It is surrounded by low to medium-sized mountains on the north, south, and east sides. The central part of the basin consists of the Yang River belt plain, flanked by sloping plains on both sides. The topography is higher in the northwest and lower in the southeast. An A-B stratigraphic section was created from Anjiabao to Gujiaying, with the specific location indicated in Fig 1A. From point A to point B, the groundwater level gradually decreases, with the grain size of the aquifer transitioning from coarse to fine (Fig 1B). The lithology in the Anjiabao region is primarily medium-coarse sand, changing to gravel and medium-coarse sand in Shalingzi, and further transitioning to silty clay and fine silt beyond Xuanhua. Vertical variations in lithology are not significant. The Zhangxuan Basin has limited lacustrine sedimentation, primarily consisting of middle to upper Pleistocene and Holocene strata (Fig 2). The predominant lithology includes gravel and sand, with a small amount of clay. In certain mountainous regions, volcanic rock, dolomite, gneiss, and sandstone are sporadically distributed.

Loose rock porous aquifers in the area are well-developed and have good water yield properties. Additionally, there are limited sources of bedrock groundwater, including fissure groundwater in volcanics, pore-fissure groundwater in clasolite, and fractured karst groundwater in carbonatite. Bedrock groundwater is primarily found in regions of middle to low mountains or low hilly areas, where the water yield properties are relatively weak. This research mainly focuses on loose rock pore groundwater. The primary sources of groundwater recharge are atmospheric precipitation, followed by infiltration from river water and seepage at valley entrances. Groundwater discharge mainly occurs through artificial extraction, along with evaporation and lateral runoff downstream. One of the primary methods of artificial extraction in groundwater source field is the extraction-based approach. The area contains eight groundwater source fields, which include Jijiabang, Yaozhanbao, Yangtai, Hezixi, Shalingzi, Fuquan Water Plant, Diliutun, and Anjiabao. With the exceptions of Fuquan Water Plant, Liutun, and Anjiabao, the remaining groundwater source fields are located in the middle and lower reaches of the area. These groundwater source fields consist of multiple well clusters that extract groundwater through pumping wells. Among them, Yaozhanbao primarily provides water to the urban area of Zhangjiakou, Fuquan Water Plant serves the central urban area of Wanquan District, and Yangtai and Hezixi are the main water sources for Xuanhua District.

### 2.2 Data sources

In this study, data were collected from 103 bore wells and eight groundwater source fields (Fig 1A). All 103 bore wells are shallow groundwater wells, numbered from Z.1 to Z.103, with depths ranging from 8 to 200 meters. During the drilling process, rock cores were extracted from various depths below the surface to create borehole histograms. In this study, borehole histograms from multiple drilling sites were collected, and CAD was used to generate stratigraphic profiles (Fig 1B). Groundwater samples were collected in June of 2016, 2018, 2020, and 2022, and groundwater levels were measured in both June and December of these four years. Samples were collected in transparent plastic bottles and analyzed for Ca^2+^, Mg^2+^, K^+^, Na^+^, HCO_3_^-^, SO_4_^2-^, Cl^-^, NO_3_^-^, total hardness (TH, representing the dissolved CaCO_3_ content in groundwater), and total dissolved solids (TDS). TDS is measured using a TDS meter in the field. HCO_3_^-^and TH were determined by titration method. Ca^2+^, Mg^2+^, K^+^, and Na^+^ were analyzed using Inductively Coupled Plasma Atomic Emission Spectroscopy (ICP AES-6000), while SO₄^2^⁻, Cl^-^, and NO_3_^-^ were analyzed using Ion Chromatography (ICS-900). After the analysis of anions and cations, the charge balance error (CBE) was used to verify the analytical precision of each sample, as shown in Eq 1 [29]. The error was within an acceptable range of ±5%.

$$CBE=\frac{\sum cations-\sum anions}{\sum cations+\sum anions}\times 100\%$$ (1)

The concentrations of anions and cations were expressed in meq/L.

The field surveys and sample collection conducted in this study did not involve any protected areas, endangered species, or regions subject to special legal restrictions. All sampling sites were located on public land or in open areas, and the research activities were fully compliant with local environmental protection regulations. Therefore, no additional permits or approvals were required for the field survey and sample collection in this study.

### 2.3 Research method

This study analyzed the hydrochemical components, types, and temporal and spatial distribution characteristics of ion concentrations in shallow groundwater from 2016 to 2022 using box plots, Piper diagrams, and distribution maps of major ion concentrations. The Gibbs diagram was employed to explore the mechanisms of groundwater genesis. Correlation analysis, ion-ratio relationship diagram, and chlor-alkali index diagram were used to further investigate the formation processes and controlling factors of groundwater hydrochemistry [30,31]. Additionally, the relationship between groundwater level fluctuations and hydrochemical evolution was examined using groundwater flow field and groundwater level data from 2016 to 2022.

The Piper diagram was utilized in the analysis of groundwater chemical components to illustrate the various chemical types present in groundwater samples. This diagram was structured in a diamond shape with six distinct areas, each representing a unique combination of ions that signify different groundwater chemistry types within the samples [32,33]. It provided an intuitive understanding of the chemical characteristics of groundwater [34]. The temporal and spatial distribution characteristics of hydrochemistry were described using ion concentration distribution maps. Given that the ion concentrations at each point followed a normal distribution and ordinary kriging interpolation demonstrated higher accuracy, ordinary kriging was chosen for spatial interpolation [35,36]. Similarly, the spatial distributions of TDS and groundwater levels were also derived using ordinary kriging. Ordinary kriging was a spatial interpolation method based on Gaussian processes. According to the first law of geography, the spatial locations of samples were linked to the study variables [37]. Information from sampling points was utilized to predict the study variables at unsampled locations [38,39]. In this study, the interpolation process was conducted using ArcGIS 10.8.1 software.

Statistical methods were employed to examine the mechanisms and controlling factors involved in groundwater formation. The Gibbs diagram reflects the control of natural groundwater chemical components by three factors: atmospheric precipitation, evapotranspiration and concentration, and rock weathering. The Gibbs diagram plots two logarithmic coordinates, with the logarithm of the TDS value as the vertical coordinate and Na^+^/(Na^+^+Ca^2+^) and Cl^-^/(Cl^-^+HCO_3_^-^) as the horizontal coordinates, delineating the ranges of the three controlling factors. The sources of groundwater chemical components were determined by the locations of sampling points on the map. However, the Na^+^/(Na^+^+Ca^2+^) ratio in groundwater’s rock weathering zones exceeded the range observed in the central region of the boomerang (water-rock interaction), varying from 0.1 to 0.9 [40]. Therefore, this study refined the Gibbs model for groundwater to broaden the range of water-rock interaction. Additionally, the Gibbs diagram did not adequately account for the effects of evaporation and concentration on groundwater. Since the groundwater in this study was unaffected by evaporation and concentration, the Gibbs model can provide a preliminary analysis of the controlling factors of hydrochemistry. Correlation analysis is a statistical method used to examine relationships between two or more equivalent variables, predicting the degree of their interdependence. The correlation coefficient ranges from -1 to 1, with stronger correlations indicated by coefficients closer to 1 in absolute value [41,42]. A strong correlation between two ions in groundwater suggests a higher degree of homogeneity between them. In this study, the analysis was performed using SPSS 26. The chlor-alkali index (CAI1 and CAI2) was proposed by Schoeller [43]. CAI1 represented the ratio of the exchange of Na^+^ and K^+^ to the amount of anions produced by halite dissolution, while CAI2 represented the ratio of the exchange of Na^+^ and K^+^ to the amount of anions produced by the dissolution of other minerals in groundwater (Eqs 2 and 3). These indices together reflected the intensity of cation exchange in the chemical evolution of groundwater. An increase in the absolute value of the chlor-alkali index indicated a higher exchange of Na⁺ and K⁺, suggesting a stronger cation exchange process. A positive chlor-alkali index indicated reverse cation exchange in groundwater, involving the replacement of Na^+^ or K^+^ with Ca^2+^ or Mg^2+^ from surrounding rocks. Conversely, a negative chlor-alkali index (where the concentration of chloride ions was less than the sum of the concentrations of sodium ions and potassium ions) indicated the presence of forward cation exchange in groundwater [44].

$$CAI1=\frac{c(Cl^{‐})‐c(Na^{+})‐c(K^{+})}{c(Cl^{‐})}$$ (2)

$$CAI2=\frac{c(Cl^{‐})‐c(Na^{+})‐c(K^{+})}{c(HCO_{3}^{-})+c(SO_{4}^{2-})+c(NO_{3}^{-})+c(CO_{3}^{2-})}$$ (3)

Where: c is the milliequivalent concentration of ions, meq/L.

Finally, mathematical and geographical statistical methods were employed to create maps of groundwater flow field changes from 2016 to 2022. The correlation between groundwater level and hydrochemical evolution was then analyzed.

## 3 Results

### 3.1 Evolution of hydrochemical components and hydrochemical types

**Fig 3:**
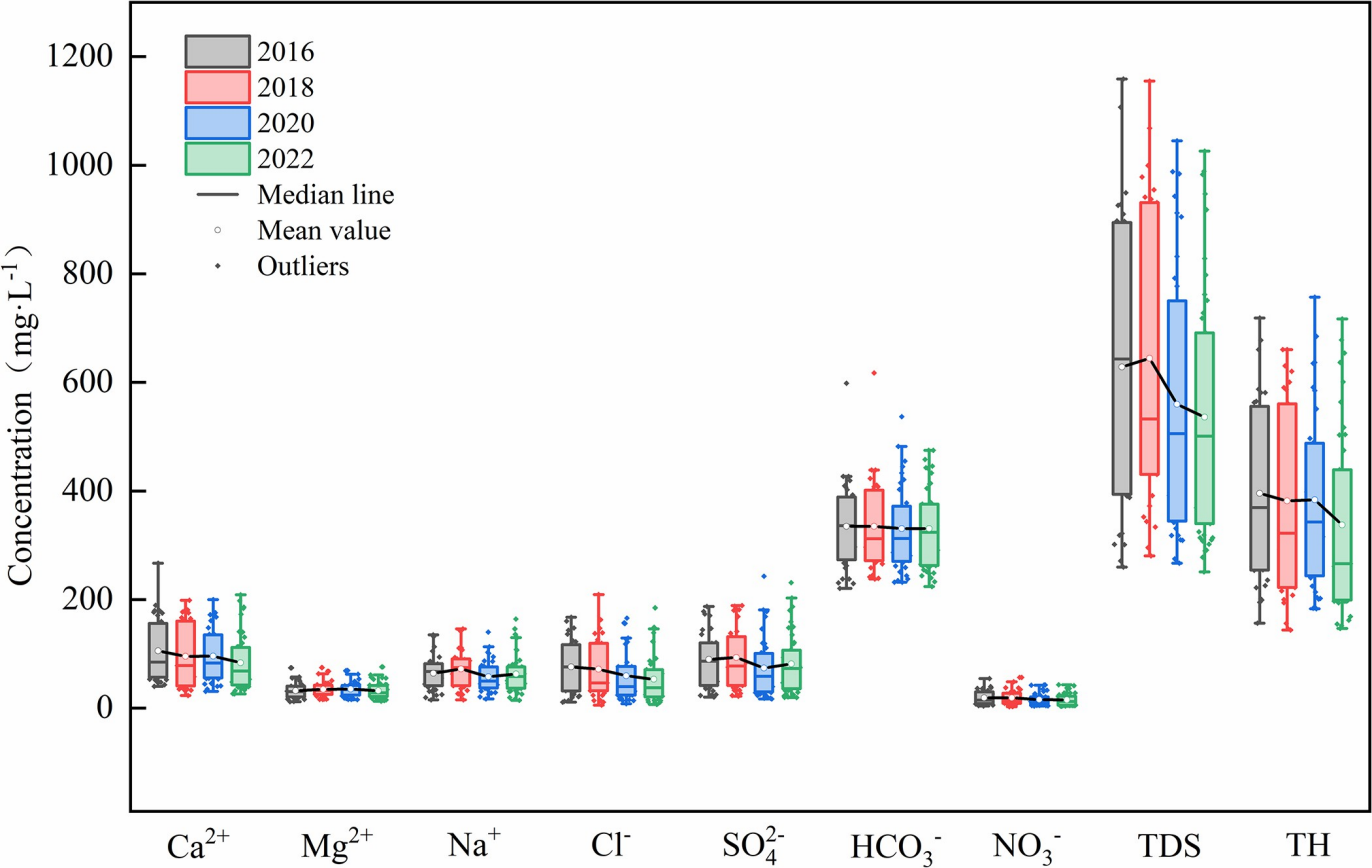
The main ionic components of groundwater in the study area.

In 2016, 2018, 2020, and 2022, the cations in shallow groundwater were primarily composed of Ca^2+^, Na^+^, and Mg^2+^, with concentrations of Ca^2+^ recorded as 105.41, 95.31, 95.72, and 83.5 mg·L^-1^, respectively. Na^+^ concentrations were 64.23, 72.2, 57.93, and 62.92 mg·L^-1^, and Mg^2+^ concentrations were 32.11, 34.90, 35.29, and 32.25 mg·L^-1^, respectively (Table 1). Between 2016 and 2022, a decreasing trend was observed for the concentrations of Ca^2+^ and Na^+^, while the concentration of Mg^2+^ remained relatively stable (Fig 3). The anions in the groundwater were primarily HCO_3_^-^, SO_4_^2-^, and Cl^-^, with concentrations of HCO_3_^-^ recorded as 334.81, 335.06, 330.50, and 330.60 mg·L^-1^, respectively. SO_4_^2-^ concentrations were 90.14, 93.21, 73.96, and 81.44 mg·L^-1^, and Cl^-^concentrations were 76.23, 71.99, 59.60, and 53.34 mg·L^-1^, respectively. From 2016 to 2022, a decline in the concentrations of HCO_3_^-^, SO_4_^2-^, Cl^-^, and NO_3_^-^ was also observed. The reduction in the concentrations of most ions in the groundwater contributed to a corresponding decrease in the concentrations of TH and TDS. The average concentration of TDS was below 1000 mg·L^-1^, indicating that the groundwater is classified as freshwater [45]. The average concentration of TH ranged from 300 to 450 mg·L^-1^, categorizing the groundwater as hard water [46].

**Table 1:** The chemical ion indicators of groundwater for 2016,2018,2020 and 2022.

Ion indicators (mg·L^-1^)		Na^+^	Ca^2+^	Mg^2+^	Cl^-^	HCO_3_^-^	SO_4_^2-^	NO_3_^-^
2016	Maximum	135.10	267.20	74.30	167.50	598.50	187.50	54.36
	Minimum	15.40	40.30	11.80	11.30	220.80	20.40	4.01
	Mean	64.23	105.41	32.11	76.23	334.81	90.14	19.31
2018	Maximum	145.91	198.80	74.86	209.19	617.49	189.10	56.49
	Minimum	15.22	23.25	16.04	5.62	237.88	22.00	2.76
	Mean	72.20	95.31	34.90	71.99	335.06	93.21	19.27
2020	Maximum	140.00	200.00	69.40	166.00	537.00	243.00	42.80
	Minimum	17.10	30.80	15.60	8.35	232.00	17.00	3.86
	Mean	57.93	95.72	35.29	59.60	330.50	73.96	15.76
2022	Maximum	164.00	209.00	76.10	185.00	475.00	231.00	43.30
	Minimum	14.50	26.40	12.90	6.97	224.00	19.70	3.45
	Mean	62.92	83.50	32.25	53.34	330.60	81.44	15.45

**Fig 4:**
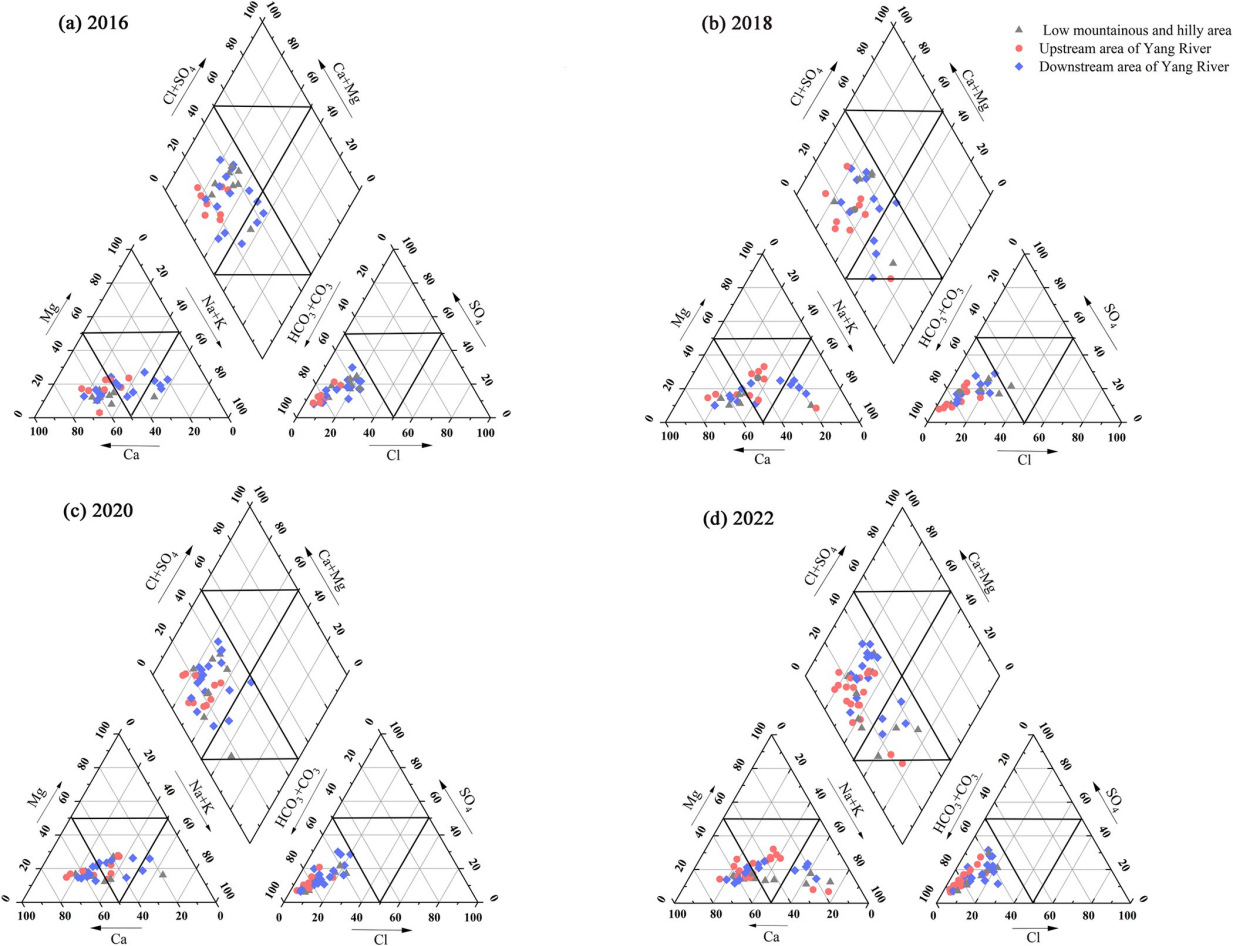
Piper trilinear diagram of groundwater in the study area.

Based on the topography and landforms, the study area was divided into three subunits: the low hilly area at the edge of the basin (mainly located in the northern and southern parts of the study area), the upper Yanghe River region in the west, and the lower Yanghe River region in the east. The hydrochemical types in these subunits showed distinct differences (Fig 4). In 2016, the predominant groundwater hydrochemical type was HCO_3_-Ca·Mg, with minor occurrences of HCO_3_-Na type groundwater in the low hilly area and the lower Yanghe River region. By 2018, the groundwater hydrochemical type remained primarily HCO_3_-Ca·Mg, but small amounts of HCO_3_-Na water were detected in the low hilly area, upper Yanghe River region, and lower Yanghe River region. In 2020, the dominant type continued to be HCO_3_-Ca·Mg, with limited HCO_3_-Na water found in the low hilly area and lower Yanghe River region. By 2022, the groundwater hydrochemical type still predominantly featured HCO_3_-Ca·Mg, while small amounts of HCO_3_-Na water were observed in all three subunits. Overall, from 2016 to 2022, the groundwater hydrochemical type across the three subunits was primarily HCO_3_-Ca·Mg, with localized areas of HCO_3_-Na type groundwater, and the distribution of HCO_3_-Na type groundwater expanded during this period. This trend indicated a deterioration in groundwater quality.

### 3.2 Spatial-temporal distribution characteristics of hydrochemistry

**Fig 5:**
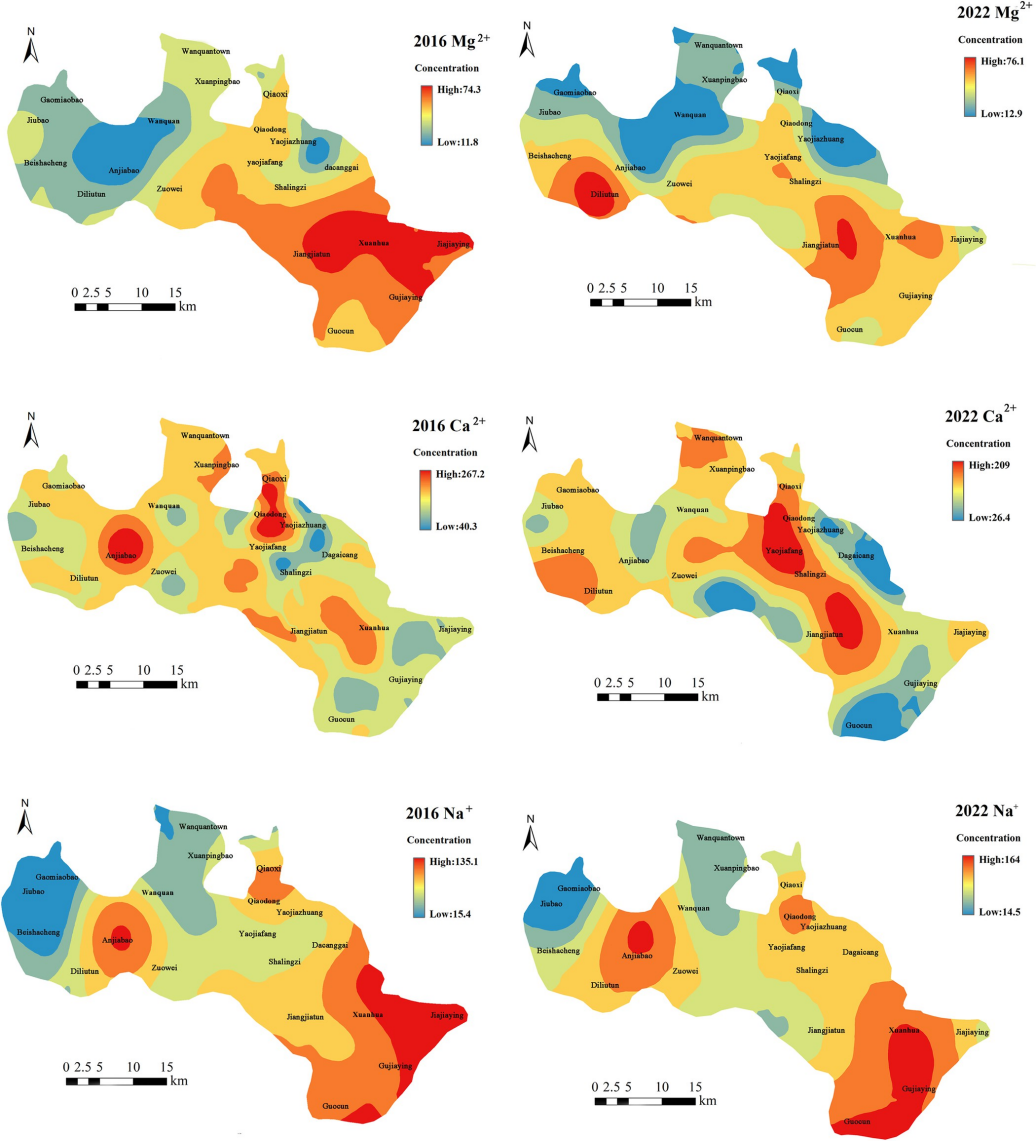
The distribution of major cations concentration in groundwater in the study area.

**Fig 6:**
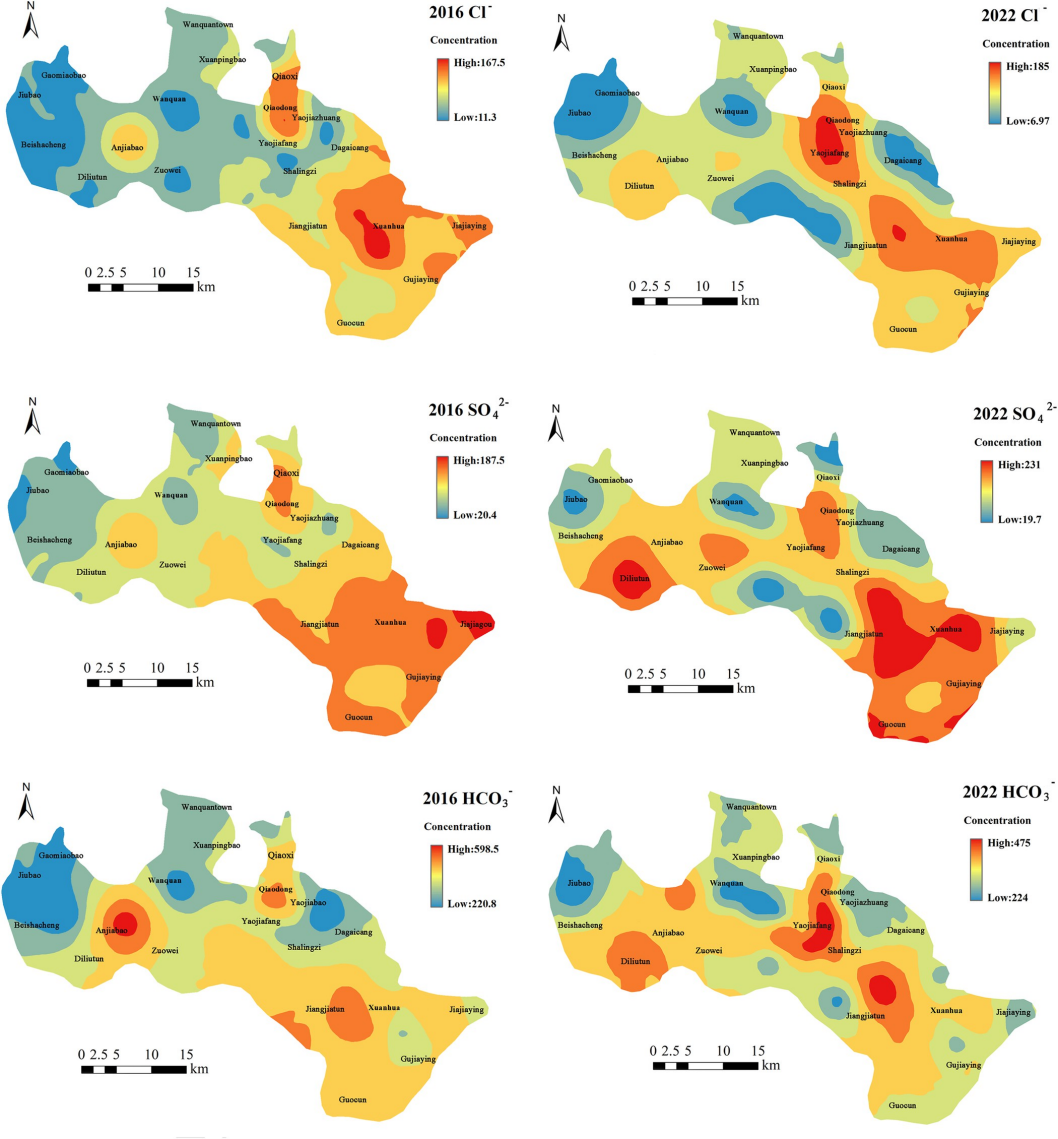
The distribution of major cations concentration in groundwater in the study area.

From 2016 to 2022, the concentrations of all major ions gradually increased along the groundwater flow direction from the northwest to the southeast (Figs 5 and 6). In 2016, the concentration of Mg^2+^ ranged from 11.8 to 74.3 mg·L^-1^. Lower concentrations were observed in the low mountainous and hilly areas and in the upstream region of the Yanghe River, while higher concentrations were found downstream, particularly in Jiangjiatun and Xuanhua. By 2022, the concentration range of Mg^2+^ had increased to 12.9 to 76.1 mg·L^-1^. A notable increase in Mg^2+^ concentration was recorded in Diliutun, upstream of the Yanghe River, compared to 2016. In 2016, the concentrations of Ca^2+^ varied from 40.3 to 267.2 mg·L^-1^. Lower concentrations were found in the low mountainous and hilly areas and the upstream Yanghe River region, except for Anjiabao, Qiaoxi, and Qiaodong, where concentrations were unusually high. Higher concentrations were observed downstream, particularly in Xuanhua. In 2022, the concentrations of Ca^2+^ ranged from 26.4 to 209 mg·L^-1^. Compared to 2016, in the upstream Yanghe River region, Ca^2+^ concentrations decreased in Anjiabao but increased near Diliutun. In the downstream Yanghe River region, the distribution range of high Ca^2+^ concentrations expanded downstream, notably at Yaojiafang. The concentrations of Na^+^ in 2016 ranged from 15.4 to 135.1 mg·L^-1^. Most low mountainous and hilly areas had lower concentrations, except in Guocun. Similarly, Na^+^ concentrations were lower in the upstream Yanghe River region, except in Anjiabao, Qiaoxi, and Qiaodong, where concentrations were unusually high. Higher Na^+^ concentrations were recorded downstream, especially in Jajiaying. By 2022, Na^+^ concentrations ranged from 14.5 to 164 mg·L^-1^, showing no significant change from 2016.

In 2016, the concentrations of Cl^-^ ranged from 11.3 to 167.5 mg·L^-1^. Lower concentrations were observed in the low mountainous and hilly areas and in the upstream Yanghe River region, except for Anjiabao, Qiaoxi, and Qiaodong. Elevated Cl^-^ concentrations were recorded downstream, particularly in Xuanhua. By 2022, Cl^-^ concentrations ranged from 6.97 to 185 mg·L^-1^. Compared to 2016, Cl^-^ concentrations in Anjiabao in the upstream Yanghe River region decreased, while those in Diliutun increased. Other regions showed minimal changes. The concentrations of SO_4_^2-^ in 2016 ranged from 20.4 to 187.5 mg·L^-1^. Lower concentrations were found in the low mountainous and hilly areas, except in Guocun, and in the upstream Yanghe River region, except in Anjiabao, Qiaoxi, and Qiaodong. Higher SO_4_^2-^ concentrations were recorded downstream, particularly in Jajiaying. By 2022, SO_4_^2-^ concentrations ranged from 19.4 to 231 mg·L^-1^. Compared to 2016, SO_4_^2-^ concentrations in Diliutun in the upstream Yanghe River region increased, while those in Anjiabao returned to normal. In the downstream Yanghe River region, concentrations in Jiangjiatun and Xuanhua increased, whereas the concentration in Jiajiaying decreased. HCO_3_^-^ concentrations in 2016 ranged from 220.8 to 598.5 mg·L^-1^. Lower concentrations were observed in the low mountainous and hilly areas and in the upstream Yanghe River region, except in Anjiabao and Qiaodong. Higher concentrations were found downstream, particularly in Jiangjiatun. By 2022, HCO_3_^-^ concentrations ranged from 224 to 475 mg·L^-1^. Compared to 2016, the concentrations of HCO_3_^-^ in Diliutun in the upstream Yanghe River region increased, while those decreased in Anjiabao. In the downstream Yanghe River region, the distribution range of high HCO_3_^-^ concentrations expanded, with a significant increase observed in Yaojiafang. From 2016 to 2022, Na^+^ and SO_4_^2-^ concentrations in Guocun, located in the low mountainous and hilly areas, remained consistently high. In the upstream Yanghe River region, ion concentrations exhibited significant changes. In 2016, Anjiabao had high concentrations of all ions except Mg^2+^, whereas in 2022, Diliutun exhibited high concentrations for all ions. The downstream Yanghe River area showed fewer significant changes, with higher ion concentrations primarily observed in Jiangjiatun, Xuanhua, and Jajiaying.

### 3.3 Source analysis of hydrochemical components

#### 3.3.1 Rock weathering

**Fig 7:**
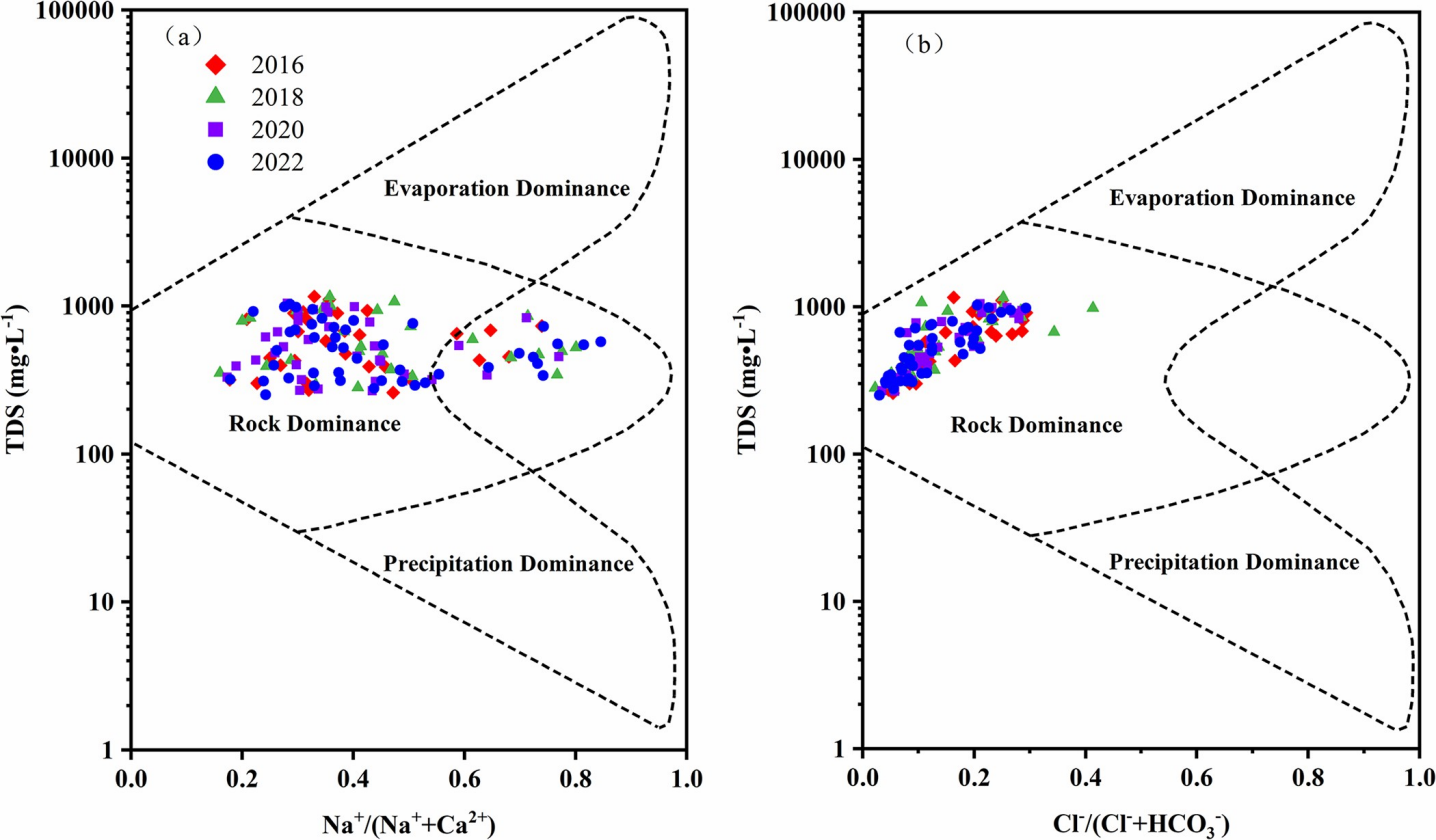
Gibbs diagram of groundwater.

The factors controlling groundwater components were categorized into evaporation control, rock weathering control, and atmospheric precipitation control (Fig 7). The ratios of Na^+^/(Na^+^+Ca^2+^) and Cl^-^/(Cl^-^+HCO_3_^-^) ranged from 0.16 to 0.85 and 0.02 to 0.41, respectively. The TDS concentration ranged from 251.0 to 1158.8 mg·L^-1^. All sampling points from 2016, 2018, 2020, and 2022 fell within the rock weathering control zone, indicating that the chemical components of groundwater were primarily influenced by rock weathering dissolution [47]. Furthermore, some sampling points lay outside the model boundaries, suggesting that human activities may have also affected the chemical composition of groundwater.

**Fig 8:**
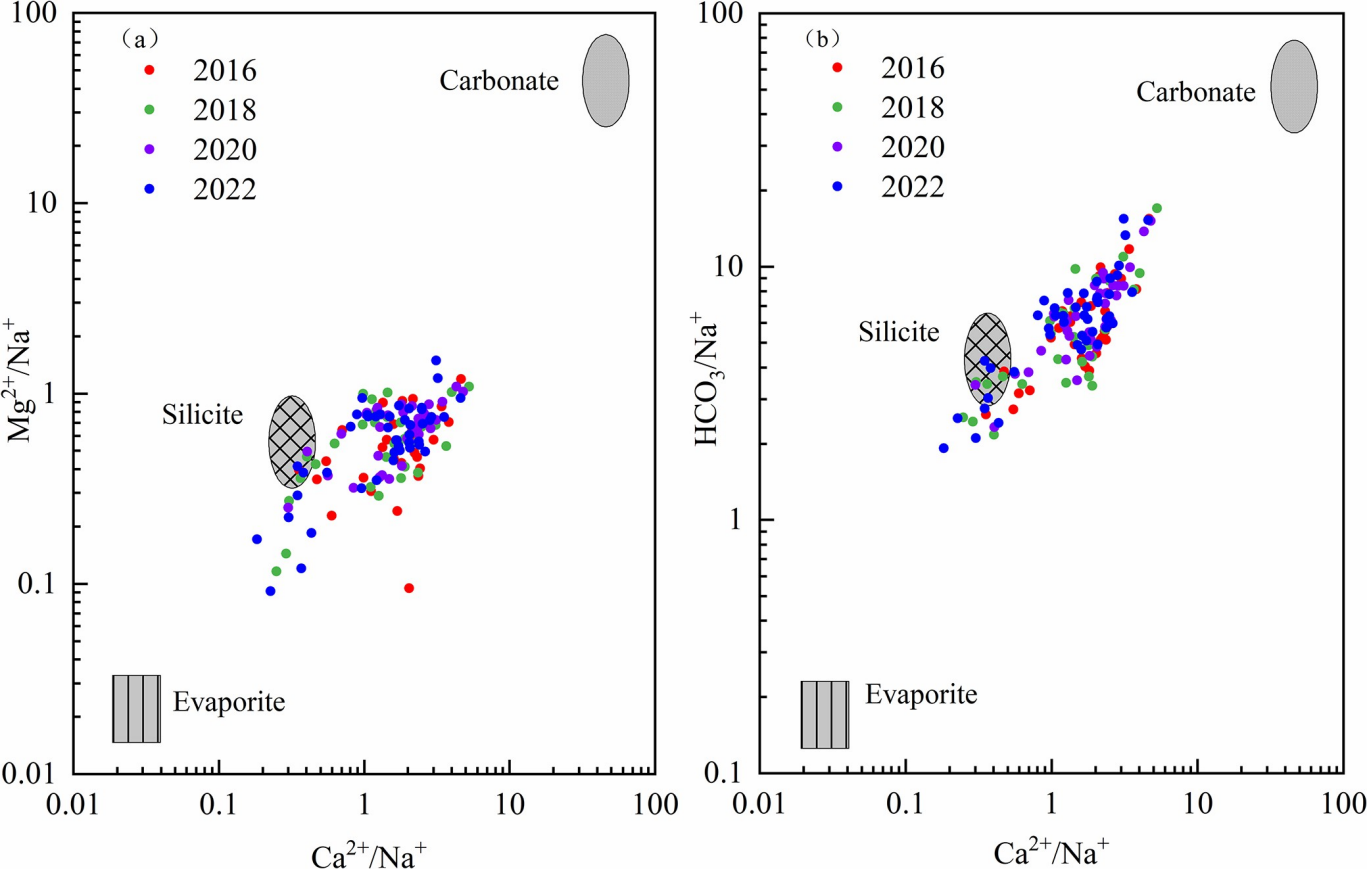
Endmember diagram of groundwater rock weathering.

The relationships between the Mg^2+^/Na^+^, Ca^2+^/Na^+^, and HCO_3_^-^/Na^+^ ratios were used to explain the interactions between groundwater and various rock types [48]. Carbonate rocks primarily contain minerals such as dolomite and calcite, which are rich in Mg^2^⁺, Ca^2^⁺, and HCO₃⁻. When the ratios of Mg^2+^/Na^+^, Ca^2+^/Na^+^, and HCO_3_^-^/Na^+^ exceed 10, it indicates significant weathering of carbonate rocks. Silicate rocks contain abundant Na^+^. When the weathering of these rocks is pronounced, the Ca^2+^/Na^+^ and Mg^2+^/Na^+^ ratios decrease, falling within the range of 1 to 10. In evaporitic rocks, which are abundant in Na^+^ and Cl^-^, the ratios of Mg^2+^/Na^+^, Ca^2+^/Na^+^, and HCO_3_^-^/Na^+^ fall below 1, suggesting that weathering of evaporitic rocks is dominant. The ratios of Ca^2+^/Na^+^, Mg^2+^/Na^+^ and HCO_3_^-^/Na^+^ ranged from 0.18 to 5.27, 0.09 to 1.49, and 1.92 to 17.00, respectively (Fig 8). Most sampling points from 2016, 2018, 2020, and 2022 were located between the weathering endmembers of silicate and carbonate rocks, while a few sampling points from 2018 and 2022 were found between the weathering endmembers of silicate and evaporite. This indicated that groundwater was primarily influenced by the weathering of silicate and carbonate rocks (Eqs 4 and 5), with minimal impact from evaporite.


$$NaAlSi_{3}O_{8}+CO_{2}+2H_{2}O=+3SiO_{2}+NaHCO_{3}+Al{\left(OH\right)}_{3}$$ (4)

$$CaCO_{3}+CO_{2}+H_{2}O\to Ca^{2+}+2HCO_{3}^{-}$$ (5)

#### 3.3.2 Leaching

The major ion concentrations in groundwater followed a normal distribution and exhibited linear correlations. Therefore, Pearson correlation analysis was conducted [49]. The correlation coefficients between Ca^2+^, Cl^-^, HCO_3_^-^, SO_4_^2-^, and TDS exceeded 0.8, indicating a strong positive correlation (Table 2). This suggested that these ions significantly contributed to TDS in groundwater. The correlation coefficient between K^+^ and TDS was very low due to the extremely low concentration of K^+^, which constituted less than 1% of the total cations and anions. Consequently, the contribution of K^+^ to TDS was negligible. The Pearson correlation coefficient between Na^+^ and Cl^-^ exceeded 0.5, indicating a shared source, potentially arising from the dissolution of halite [50]. Ca^2+^ showed a significant correlation with HCO_3_^-^, suggesting that these ions originated from the dissolution of dolomite and calcite. Additionally, Ca^2+^ exhibited a significant correlation with Cl^-^, while SO_4_^2^ showed a notable correlation with Na^+^, Ca^2+^, and Mg^2+^. This association was attributed to the dissolution of gypsum and dolomite [51].

**Table 2:** Correlation analysis of groundwater hydrochemical components.

	K^+^	Na^+^	Ca^2+^	Mg^2+^	Cl^-^	HCO_3_^-^	SO_4_^2-^	TDS	TH
K^+^	1								
Na^+^	0.027	1							
Ca^2+^	0.068	0.203*	1						
Mg^2+^	0.181	0.409**	0.365**	1					
Cl^-^	0.135	0.521**	0.789**	0.596**	1				
HCO_3_^-^	0.135	0.537**	0.776**	0.561**	0.653**	1			
SO_4_^2-^	0.163	0.684**	0.624**	0.748**	0.742**	0.745**	1		
TDS	0.196	0.608**	0.855**	0.659**	0.896**	0.864**	0.887**	1	
TH	0.122	0.314**	0.940**	0.660**	0.855**	0.831**	0.776**	0.932**	1

**Fig 9:**
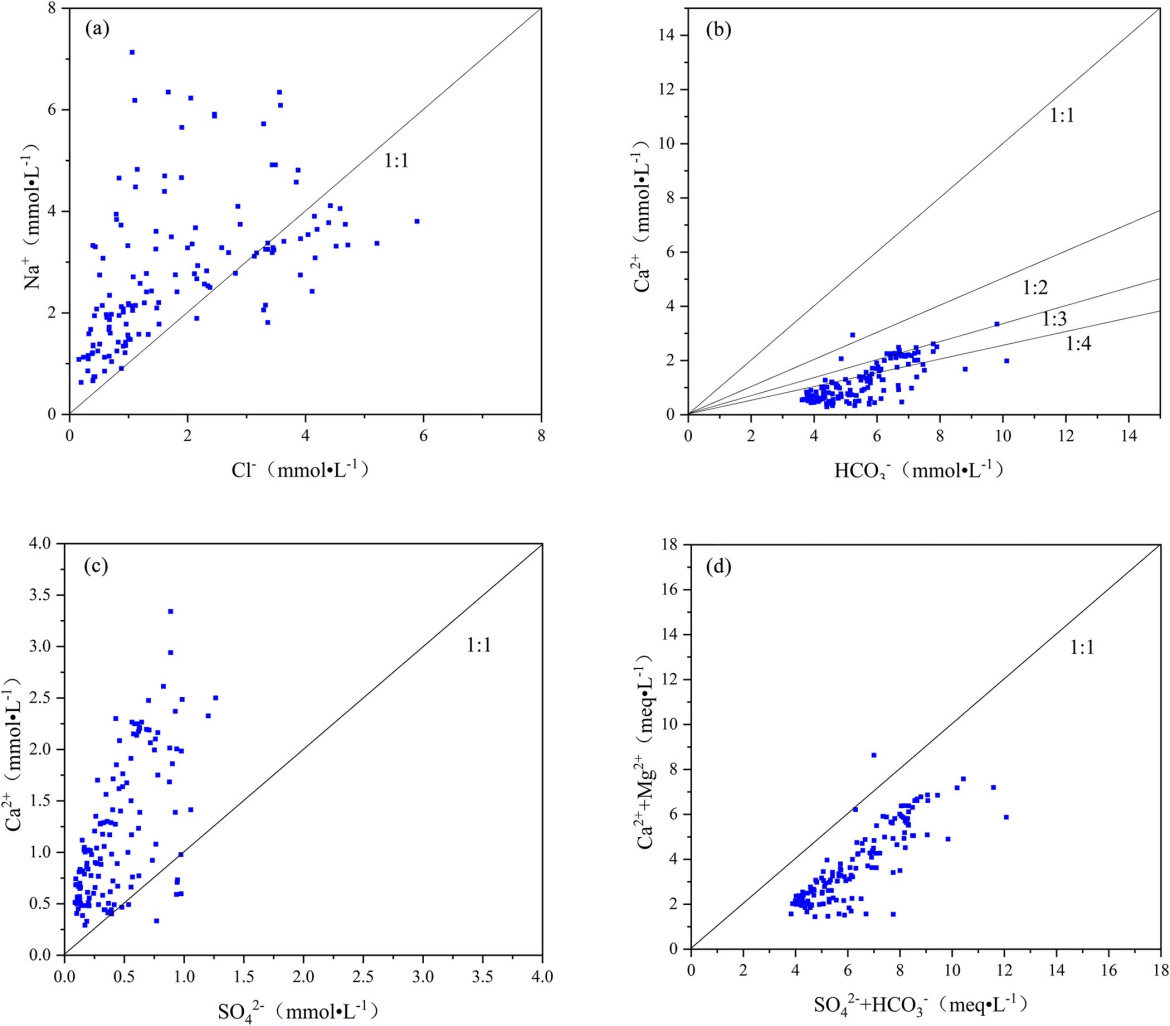
Ionic relationships of groundwater in the study area.

Under natural circumstances, the dissolution of halite released equal amounts of Na^+^ and Cl^-^ into groundwater. If the Na^+^ in groundwater is primarily derived from the dissolution of halite, the Na^+^/Cl^-^ ratio will approach 1:1, as indicated in Eq 6 [52]. Most sampling points exhibited a Na^+^/Cl^-^ ratio greater than 1 (Fig 9A), with Na^+^ concentrations exceeding those of Cl^-^. This finding indicated that, in addition to halite dissolution, forward cation exchange processes and the dissolution of sodium-rich silicate minerals, such as feldspar. This finding was consistent with the previous discussion about the influence of silicate rocks on groundwater. Furthermore, a small number of scattered points were found below the 1:1 line, where Na^+^ concentrations decreased, possibly due to forward cation exchange processes that reduced sodium ion concentrations.


$$NaCl\to Na^{+}+Cl^{-}$$ (6)

Correlation analysis revealed a significant correlation between Ca^2+^ and HCO_3_^-^, indicating that groundwater was greatly influenced by carbonates. Calcite and dolomite, the primary minerals in carbonate rocks, dissolve to produce varying proportions of Ca^2+^ and HCO_3_^-^. Therefore, an analysis of these two minerals was performed. In groundwater, when only calcite dissolution occurred, a Ca^2+^/HCO_3_^-^ ratio of 1:2 was produced (Eq 5). When only dolomite dissolution occurred, a Ca^2+^/HCO_3_^-^ ratio of 1:4 was generated (Eq 7). When both dolomite and calcite dissolved simultaneously, a Ca^2+^/HCO_3_^-^ ratio of 1:3 was observed. Most sampling points fell below the 1:2 line, with some located between 1:2 and 1:4 (Fig 9B). This indicated that a portion of Ca^2+^ and Mg^2+^ in groundwater originated from the dissolution of carbonates dominated by dolomite and calcite, confirming that the spatial and temporal distribution of Ca^2+^ and HCO_3_^-^ was influenced by carbonate dissolution. Additionally, some sampling points were positioned below the 1:4 line, where the concentration of Ca^2+^ decreased due to forward cation exchange processes. Most sampling points were located near and above the 1:1 line (Fig 9C), indicating that SO₄^2-^ in groundwater originated from the dissolution of gypsum, while Ca^2^⁺ came not only from gypsum dissolution but also from the dissolution of other minerals. The ratio of (Ca^2+^+Mg^2+^)/(SO_4_^2-^+HCO_3_^-^) was further analyzed. Most sampling points were found below the line, with a few scattered points above it (Fig 9D), further indicating that Ca^2+^ and Mg^2+^ were primarily influenced by the dissolution of dolomite and calcite, while also being affected by cation exchange.


$$CaMg{\left(CO_{3}\right)}_{2}+2CO_{2}+2H_{2}O\to Mg^{2+}+Ca^{2+}+4HCO_{3}^{-}$$ (7)

#### 3.3.3 Ion exchange

**Fig 10:**
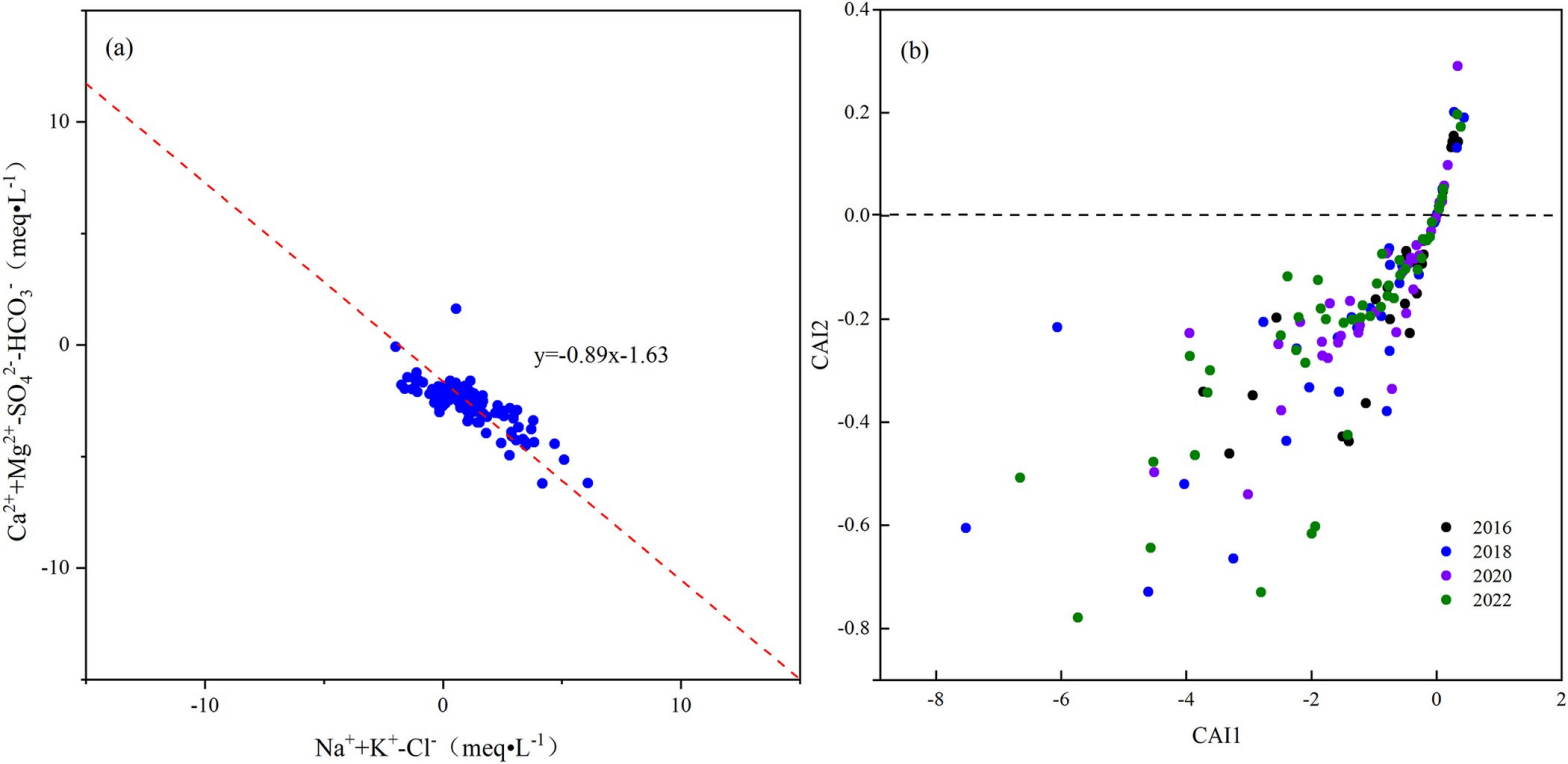
Cation exchange and adsorption of groundwater in the study area.

Cl^-^, SO_4_^2-^, and HCO_3_^-^ in groundwater were driven by the dissolution of halite, potassium salt, calcite, dolomite, and gypsum. The value of (Na⁺+K^+^-Cl^-^) indicated the amount of Na⁺ that increased or decreased as a result of the dissolution of halite and potassium salt. The value of (Ca^2+^+Mg^2+^-SO_4_^2—^HCO_3_^-^) reflected the changes in Ca^2+^ and Mg^2+^ due to the dissolution of gypsum, calcite, and dolomite. If a linear relationship was found between these two parameters with a slope of ‘-1’, it indicated that cation exchange reactions occurred in groundwater. Most sampling points were located near the line y = -0.89x-1.63, with a slope approaching -1 (Fig 10A). This indicated that Na⁺, Ca^2+^, and Mg^2+^ participated in cation exchange processes in the groundwater, influenced by the dissolution of silicate and carbonate rocks.

The chlor-alkali index was further utilized to investigate cation exchange during the groundwater transport process. The relationship between CAI1 and CAI2 was analyzed (Fig 10B). The values of CAI1 ranged from -7.52 to 0.44, while CAI2 ranged from -0.78 to 0.29. Notably, 83.1% of the sampling points had CAI1 and CAI2 values less than 0, while 16.9% had values greater than 0. This finding suggested that forward cation exchange predominated, whereas positive cation exchange was relatively weak. In most parts of the study area, Ca^2+^ and Mg^2+^ in the groundwater were exchanged with Na⁺ and K⁺ from the surrounding formations, resulting in increased concentrations of Na⁺ and K⁺ in the groundwater. In some areas, Na⁺ and K⁺ in the groundwater exchanged with Ca^2+^ and Mg^2+^ from the surrounding formations, leading to increased concentrations of Ca^2+^ and Mg^2+^ in the groundwater.

## 4 Discussion

### 4.1 Spatial-temporal evolution of groundwater level

**Fig 11:**
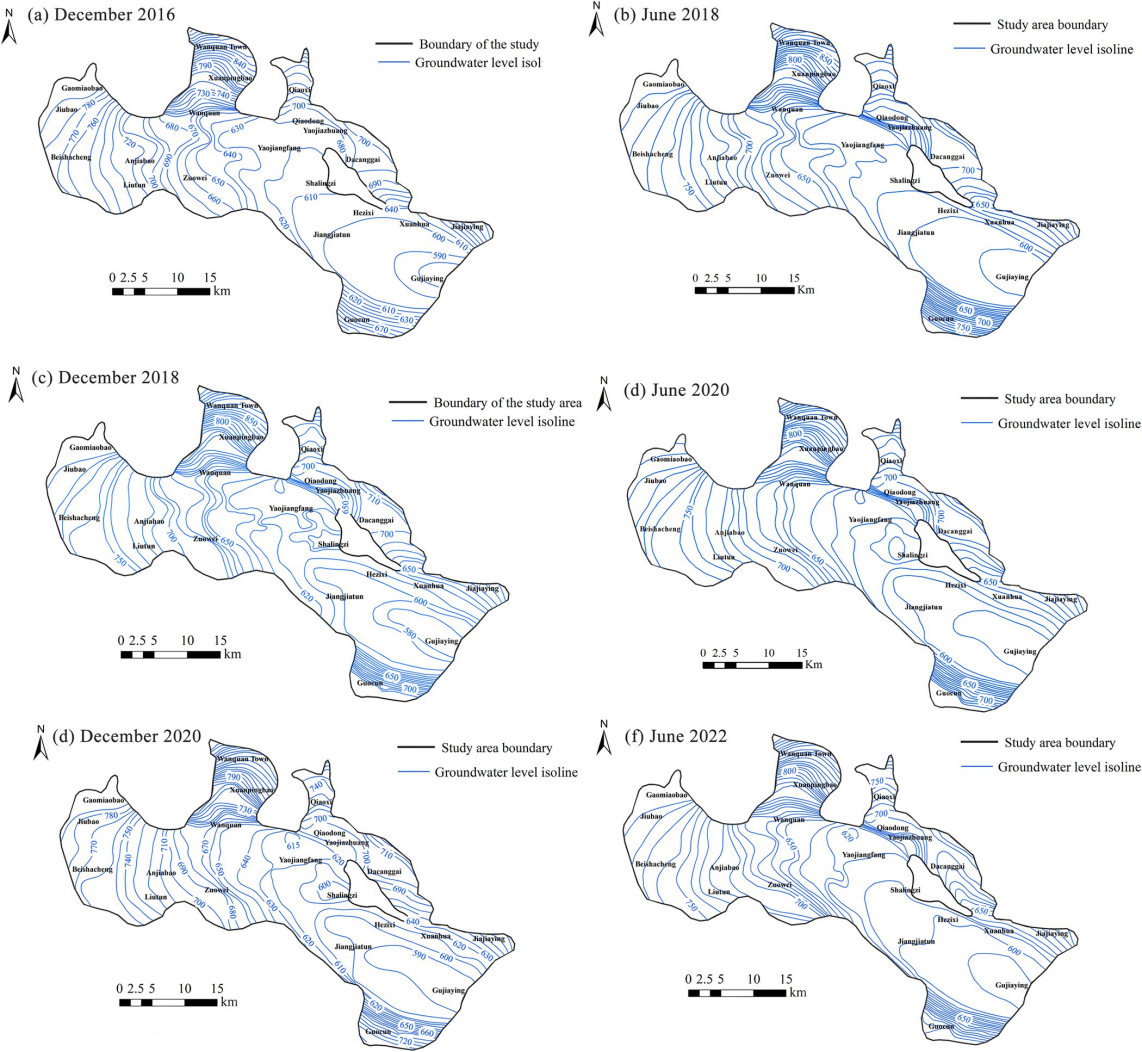
Groundwater contour map of the study area.

The general direction of groundwater flow remained consistent from 2016 to 2022, moving from the northwest to the southeast (Fig 11). The highest groundwater level in the study area was observed in Wanquan Town in the north, while the lowest groundwater level was recorded in Gujiaying in the southeast. From December 2016 to June 2018, the groundwater level in the western region remained stable, while significant fluctuations occurred in the east. Qiaoxi and Qiaodong, situated in hilly mountainous areas, experienced a 10-meter rise in groundwater level due to recharge from mountain-front runoff. In Guocun, the groundwater level increased by 50 meters. On one hand, this was due to the groundwater flowing through a fine sand layer with low permeability, resulting in minimal downstream flow. On the other hand, it was influenced by recharge from upstream runoff. Gujiaying also experienced a 10-meter rise from upstream recharge. Conversely, the southern part of Yaojiafang saw a decline in groundwater level due to the Yaozhanbao groundwater source fields. From June to December 2018, the groundwater level in southern Yaojiafang continued to decline by about 5 meters due to influences from the Yaozhanbao groundwater source fields. Shalingzi and Jiangjiatun experienced a similar decrease of approximately 10 meters in groundwater level, attributed to the Shalingzi and Yangtai groundwater source fields. Gujiaying also saw a decrease of about 10 meters in groundwater level influenced by upstream factors. From December 2018 to June 2020, Yaojiafang and Anjiabao experienced a 10-meter decline in groundwater level due to extraction from groundwater source fields. In the eastern region of Anjiabao, the groundwater level dropped by approximately 10 meters because of reduced upstream recharge and river seepage. Gujiaying’s groundwater level, supported by upstream recharge, showed an upward trend. From June to December 2020, Gujiaying and its surroundings experienced declining groundwater level, attributed to the Hezixi groundwater source fields. From December 2020 to June 2022, groundwater level in the eastern mountainous region continued to decline, while those in the southeastern plains began to recover. In Shalingzi, groundwater level rose by 10 meters following water management efforts in Zhangjiakou in 2022, which curtailed groundwater extraction.

In 2018 and 2020, from the premonsoon (June) to the postmonsoon (December), overall groundwater levels did not recover, indicating minimal recharge from precipitation. Based on the direction of groundwater flow, Jiubao, Wanquan Town, and Guocun in the upstream areas of the Yanghe River were identified as recharge areas, while Gujiaying in the downstream area was identified as a discharge area. The groundwater level in the recharge area increased, accompanied by a gradual rise in the hydraulic gradient. In the discharge area, the groundwater level gradually declined but showed recovery after 2020.

### 4.2 Influence of groundwater flow on the hydrochemical characteristics

From 2016 to 2022, groundwater anions and cations were consistently dominated by HCO_3_^-^ and Ca^2+^. The predominant groundwater chemistry type was HCO_3_-Ca·Mg. This was primarily due to leaching processes in the groundwater, which caused the dissolution of carbonate and silicate, leading to increased concentrations of Ca^2+^, Mg^2+^, and HCO_3_^-^. The analysis of hydrochemical evolution indicated a gradual expansion of the HCO_3_-Na type groundwater. This change was driven by three main factors: (1) The rise in Na^+^ concentration due to forward cation exchange in groundwater. (2) With the flow of groundwater, the hydraulic retention time lengthened, causing extended contact with the surrounding rocks. This enhanced the leaching effect, leading to a rise in Na^+^ concentration. (3) Groundwater flow from the northwest to the southeast caused a lithological shift in the aquifer from gravel to silty clay. Additionally, the decline in groundwater levels in the southeastern discharge area further shifted the aquifer to silty clay. The interaction between clay and groundwater promoted forward cation exchange, leading to an increase in sodium ions and HCO_3_-Na type groundwater.

The spatial distribution of hydrochemical components exhibited a trend of increasing concentrations from the northwest to the southeast. In the recharge area in the northwest, the steeper hydraulic gradient, faster groundwater flow, and shorter hydraulic retention time reduced the leaching effect, resulting in lower concentrations of Na^+^, Ca^2+^, Mg^2+^, Cl^-^, SO_4_^2-^, and HCO_3_^-^ compared to the southeastern discharge area. Localized increases in hydrochemical concentrations were observed in areas such as Anjiabao and Diliutun in the upstream region of the Yanghe River, and Jijiafang, Shalingzi, and Hezixi downstream. These areas contained groundwater source fields, and extensive groundwater extraction led to a decline in groundwater level. In Anjiabao, the groundwater level decreased by 10 meters from 2018 to 2020, while in Xuanpingbao, it fell by 20 meters from 2018 to 2022. Additionally, a groundwater funnel formed in Shalingzi between 2018 and 2020. The decline in groundwater levels caused groundwater to converge toward the centers of the groundwater source fields, leading to a gradual enrichment of ionic components and a significant increase in ion concentrations.

Changes in groundwater levels altered aquifer lithology, which in turn affected ion concentrations. From 2016 to 2022, the Anjiabao aquifer consisted mainly of fine silt sand, which was prone to forward cation exchange, leading to a reduction in Mg^2+^ and an increase in Na^+^. In Anjiabao and southern Xuanhua, the concentrations of Ca^2+^ and HCO_3_^-^ decreased significantly over time, indicating carbonate rock dissolution. However, as groundwater level dropped, the contact area between groundwater and surrounding rocks diminished, weakening the leaching effect.

As groundwater level rose, both TDS and TH decreased. The hydrochemical composition was evidently influenced by the weathering and dissolution of silicate and carbonate rocks. As groundwater depth increased, the interaction between groundwater and surrounding rocks diminished, leading to reduced ion dissolution and consequently lower TDS. According to the A-B stratigraphic section, there was a decrease in water level in the discharge area. Groundwater transitioned from silt layers to silt-clay layers, which reduced Ca^2+^ concentrations due to forward cation exchange. The concentration of Mg^2+^ remained constant, resulting in a decrease in TH. The impact of groundwater depth on groundwater chemistry was significant, affecting water-rock interactions and solute migration.

**Fig 12:**
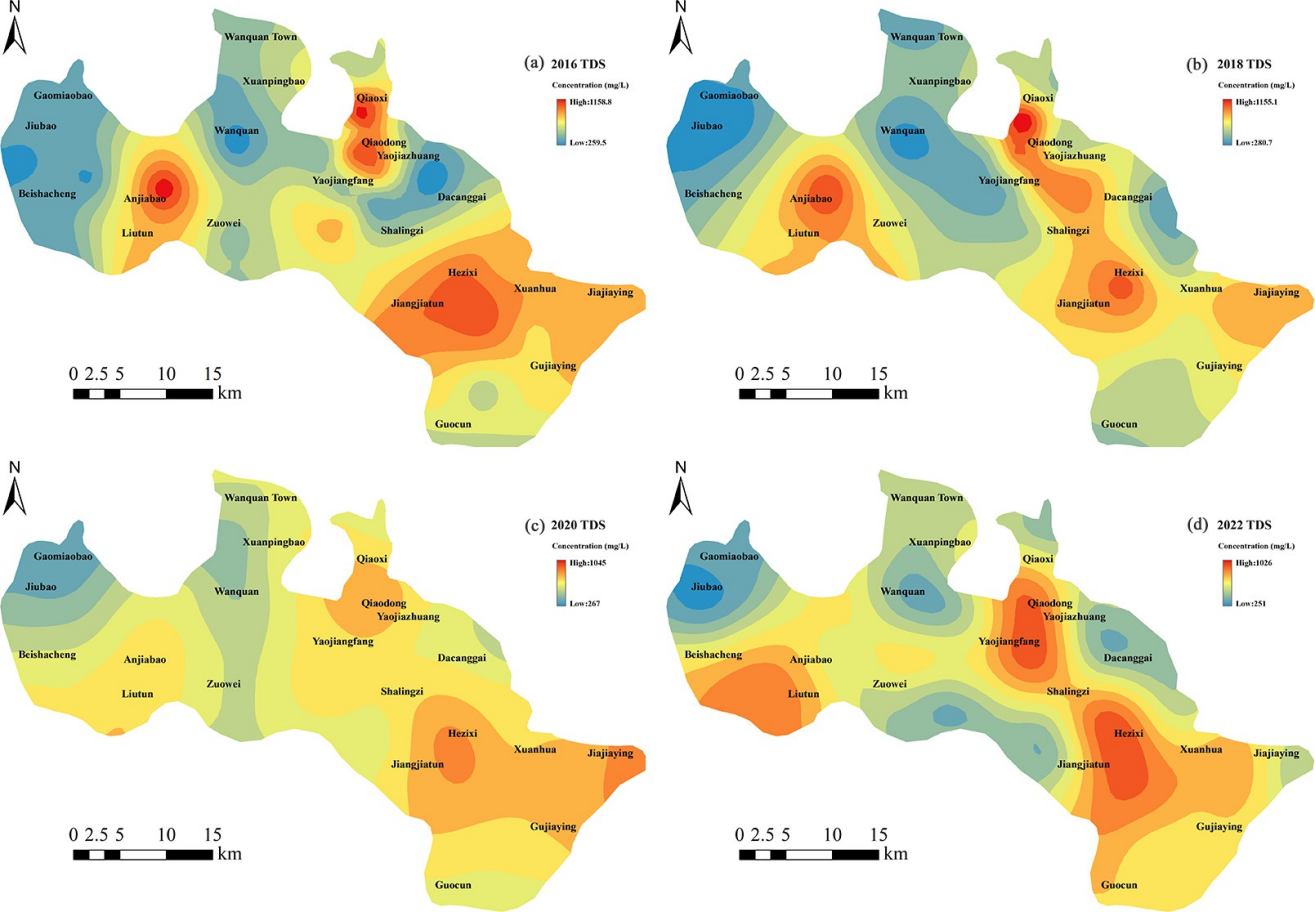
The distribution of TDS in groundwater in the study area.

A detailed analysis of the spatial distribution of TDS was conducted (Fig 12). From the recharge to discharge areas, TDS followed a pattern of being lower in the northwest and higher in the southeast, consistent with the direction of groundwater flow. In regions such as Anjiabao, Qiaoxi, Qiaodong, Hezixi, and Jiajia Ying, TDS remained consistently high due to extensive groundwater extraction, which accelerated groundwater flow and caused ion accumulation, leading to increased TDS. From 2016 to 2018, the groundwater level in Yaojiafang declined, resulting in a reduction in TDS. Between 2018 and 2022, in the recharge area, the groundwater level in Anjiabao declined, causing a reduction in TDS. Meanwhile, in Guocun, the groundwater level rose, leading to an increase in TDS. From 2018 to 2020, the groundwater level in the Hezixi, located in the discharge zone, decreased, leading to a reduction in TDS. In 2022, as groundwater levels rose, TDS increased accordingly. Spatially, the lithology in the recharge area was primarily composed of gravel and sand layers, while in the discharge area, the lithology became finer with higher clay content, resulting in slower groundwater flow. As groundwater moved from the recharge to discharge areas, the contact time and surface area between groundwater and the surrounding rock increased, enhancing leaching and introducing more ions into the groundwater. Additionally, groundwater flow caused ion accumulation in the discharge area, further increasing TDS. Over time, as groundwater levels dropped, the reduced contact between groundwater and surrounding rocks weakened the leaching effect, leading to a decrease in TDS.

### 4.3 Influence of human activities

Industrial wastewater, domestic sewage, and agricultural chemicals such as pesticides and fertilizers generated by human activities infiltrated groundwater along with precipitation or surface water, increasing the concentrations of certain ions and influencing the chemical evolution of the groundwater. NO_3_^-^ is a key indicator of human impact on water bodies. In natural water, its concentration typically does not exceed 10 mg·L^-1^. However, in the study area, the average concentration of NO_3_^-^ in groundwater was 17.2 mg·L^-1^, with 61.27% of sampling points showing concentrations above 10 mg·L^-1^, indicating significant influence from human activities.

**Fig 13:**
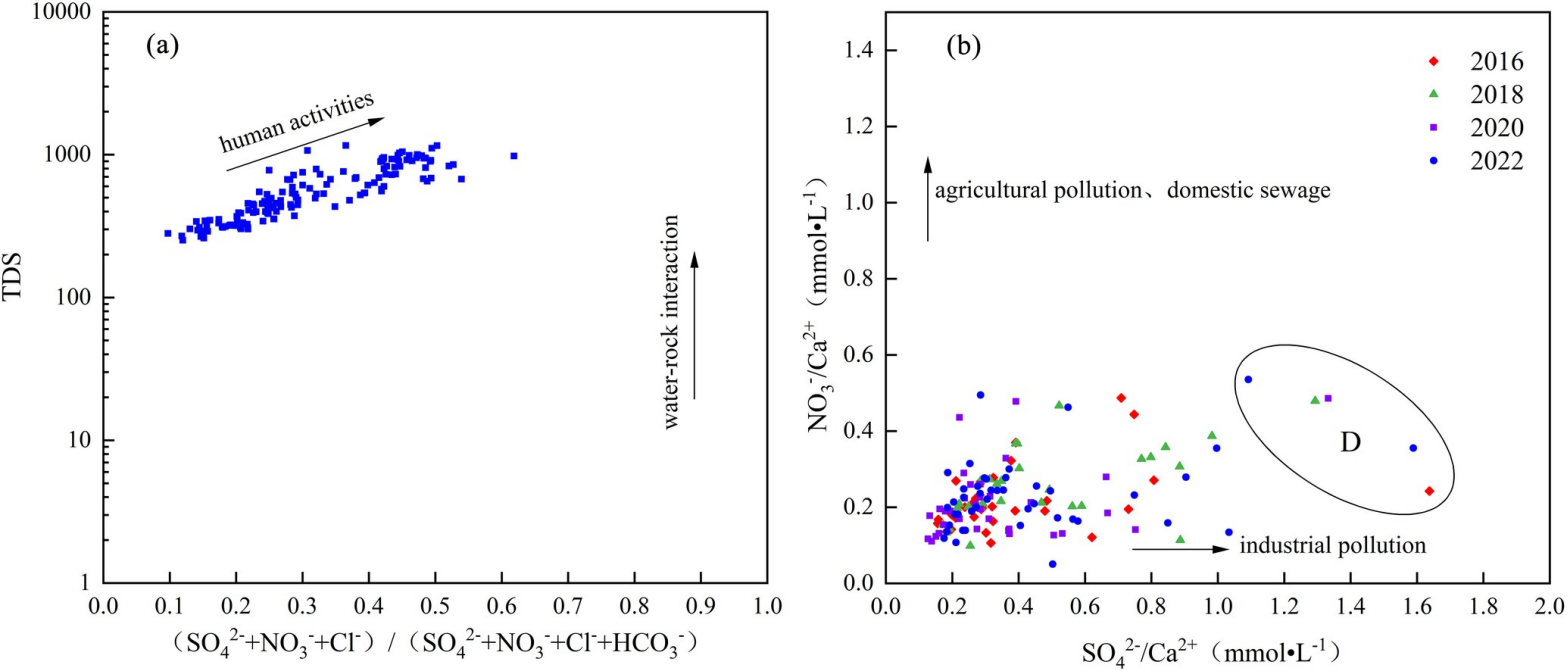
Effects of human activities on hydrochemical characteristics.

Further investigation was conducted into the relationship between TDS and (SO_4_^2-^+NO_3_^-^+Cl^-^)/(SO_4_^2-^+NO_3_^-^+Cl^-^+HCO_3_^-^), as well as between NO_3_^-^/Ca^2+^ and SO_4_^2-^/Ca^2+^. HCO_3_^-^ in groundwater was derived from water-rock interaction processes, making HCO_3_^-^ a useful indicator for assessing these interactions. When the concentration of HCO_3_^-^ was low and TDS were high, it indicated that the ions in groundwater were also affected by human activities. The spatial distribution of sampling points revealed that groundwater chemistry was influenced not only by water-rock interactions but also by human activities (Fig 13A). NO_3_^-^ primarily resulted from agricultural pollution, while SO_4_^2-^ mainly arose from industrial pollution. In the study area, the NO_3_^-^/Ca^2+^ ratio at sampling points was less than 1, while the SO_4_^2-^/Ca^2+^ ratio exceeded 1. This finding indicated that industrial activities exerted a greater influence on groundwater than agricultural pollution and domestic sewage (Fig 13B). Moreover, groundwater in area D was notably affected by industrial activities. In this area, solid waste dumps contributed pollutants that infiltrated into the groundwater through rainfall, leading to elevated concentrations of SO_4_^2-^. The presence of iron and steel industries, along with refuse landfills in the study area, could increase the levels of ions such as Na^+^, Cl^-^, and SO_4_^2-^, thereby affecting the groundwater’s chemical composition.

In areas with artificial land use, groundwater exhibited high concentrations of both cations and anions, demonstrating that human activities directly or indirectly affected groundwater chemical composition. In particular, in Qiaoxi and Qiaodong, where population density was high and groundwater demand was significant, groundwater extraction was substantial even in the absence of groundwater source fields. Additionally, the use of cement and asphalt in urban road construction reduced atmospheric precipitation recharge, which led to higher ion concentrations in these areas.

The hydrochemical composition and types were significantly influenced by fluctuations in groundwater levels and lithological stratigraphy. Leaching, cation exchange, and adsorption had particularly strong effects. Along the groundwater flow path, forward cation exchange and leaching effects increased. From 2016 to 2022, the leaching effect in groundwater decreased, while reverse cation exchange became more prominent. Human activities also contributed to changes in groundwater chemistry.

The elevated concentrations of hydrochemical components in groundwater were the result of multiple factors. Lithological formations had the greatest impact on groundwater, primarily through rock weathering, leaching, and reverse ion exchange. Industrial and agricultural activities also played a major role in altering groundwater chemistry [21]. Additionally, mixing processes affected hydrochemistry, especially in regions with geothermal groundwater [16]. However, this study did not include surface water analysis due to insufficient data. While the influence of groundwater flow on hydrochemistry was considered, limited data prevented the creation of groundwater contour maps for June 2016 and December 2022.

## 5 Conclusion

Significant changes in groundwater chemistry and levels were observed between 2016 and 2022 due to both natural factors and human activities. This study examined the chemical evolution and genetic mechanisms of shallow groundwater in the Zhangxuan Basin, China, using Piper and Gibbs diagrams, ion ratio relationships, and mathematical and statistical analysis. The conclusions were as follows:

1. The dominant cations in the study area were Ca^2^⁺, and the primary anions were HCO₃⁻. Over time, the chemical composition of groundwater showed a decreasing trend, with a gradual increase in major ion concentrations along the groundwater flow path. Some areas exhibited higher ion concentrations due to groundwater source fields and the influence of human activities.

2. The hydrochemical type was mainly HCO_3_-Ca·Mg, followed by HCO_3_-Na. Along the groundwater flow, HCO_3_-Na type groundwater increased over time, leading to a deterioration in groundwater quality. This increase was attributed to the gradual intensification of forward cation exchange and leaching processes.

3. From 2016 to 2022, groundwater chemistry was primarily influenced by leaching and forward cation exchange. The dissolution of silicate and carbonate minerals played a major role in geochemical processes. Carbonate minerals contributed Mg^2+^, Ca^2+^, and HCO_3_^-^, while Na^+^ and Cl^-^ originated from halite dissolution. Additionally, the dissolution of silicate minerals and forward cation exchange contributed to the rise in Na^+^, while SO_4_^2-^ mainly came from gypsum dissolution. Human activities also impacted groundwater chemistry.

4. Changes in groundwater levels, lithology, and human activities significantly shaped the chemical evolution of groundwater. Groundwater flow direction and levels influenced water-rock interactions and solute transport, largely by altering the lithology of aquifers. Human activities, such as groundwater extraction and industrial waste discharge, also altered the chemical composition. Leaching decreased between 2016 and 2022, while forward cation exchange became more prominent.

## Supporting information

S1 Data (XLS)
